# Load-Bearing Capacity and Retention of Newly Developed Micro-Locking Implant Prosthetic System: An In Vitro Pilot Study

**DOI:** 10.3390/ma11040564

**Published:** 2018-04-06

**Authors:** Jae-Won Choi, Kyung-Hee Choi, Hee-Jin Chae, Sung-Ki Chae, Eun-Bin Bae, Jin-Ju Lee, So-Hyoun Lee, Chang-Mo Jeong, Jung-Bo Huh

**Affiliations:** 1 Department of Prosthodontics, Dental Research Institute, Institute of Translational Dental Sciences, BK21 PLUS Project, School of Dentistry, Pusan National University, Yangsan 50612, Korea; won9180@hanmail.net (J.-W.C.); 0228dmqls@hanmail.net (E.-B.B.); ljju1112@hanmail.net (J.-J.L.); romilove7@hanmail.net (S.-H.L.); cmjeong@pusan.ac.kr (C.-M.J.); 2 Research and Development Institute, Cowellmedi Co., Ltd., Busan 46986, Korea; ckh@cowellmedi.com; 3 Research and Development Institute, Samwon Dental Medical Precise Co., Ltd., Yangsan 50603, Korea; heejin917@naver.com (H.-J.C.); csg8606@naver.com (S.-K.C.)

**Keywords:** dental implant, prosthesis, micro-locking, load-bearing capacity, retention

## Abstract

The aim of this study was to introduce the newly developed micro-locking implant prosthetic system and to evaluate the resulting its characteristics. To evaluate load-bearing capacity, 25 implants were divided into five groups: external-hexagon connection (EH), internal-octagon connection (IO), internal-hexagon connection (IH), one-body implant (OB), micro-locking implant system (ML). The maximum compressive load was measured using a universal testing machine (UTM) according to the ISO 14801. Retention was evaluated in two experiments: (1) a tensile test of the structure modifications of the components (attachment and implant) and (2) a tensile test after cyclic loading (total 5,000,000 cycles, 100 N, 2 Hz). The load-bearing capacity of the ML group was not significantly different from the other groups (*p* > 0.05). The number of balls in the attachment and the presence of a hexagonal receptacle did not show a significant correlation with retention (*p* > 0.05), but the shape of the retentive groove in the implant post had a statistically significant effect on retention (*p* < 0.05). On the other hand, the retention loss was observed during the initial 1,000,000 cycles, but an overall constant retention was maintained afterward. Various preclinical studies on this novel micro-locking implant prosthetic system should continue so that it can be applied in clinical practice.

## 1. Introduction

Implant-supported fixed dental prostheses (FDP) have been a well-established treatment option for dental treatment for the last 40 years since the development of dental implants [1]. The long-term clinical success of these FDPs are based on the advancement of surface technology, sophisticated surgical techniques, improved stability of the interface between implant, and abutment dental prosthesis, as well as osseointegration [2,3].

Methods for retaining implant-supported prostheses include screw-retained prostheses that gain stability and retention from clamping force caused by preload generated by screw extension, and cement-retained prostheses that gain stability and retention from cement [4,5]. The screw-retained prostheses have been recommended for long-span restorations because they have the advantage of retrievability as well as accessibility for maintenance and replacement [1,6]. However, screw-retained prostheses have intrinsic mechanical complications such as screw loosening and fractures [5,7]. The cement-retained prostheses were recommended as a single-span restoration with a margin above the mucosal level and was used to treat partial edentulous patients [1,6]. Some authors have suggested that cement-retained prostheses should be the first treatment option in consideration of esthetics [8]. In addition, the cement-retained prostheses fit a more passive prosthesis than do screw-retained prostheses. However, it is difficult to remove excess cement around the crown, and the residual cement is likely to cause inflammation around an implant [6,9].

The success rate of these prostheses is not affected by the type of maintenance [10], but both types have relative advantages and disadvantages and can affect the frequency of technical and biological complications [6,11]. Additionally, because the mechanical stability of the prosthesis is affected by the engineering of the implant–abutment interface, retention mechanisms can affect these results [12]. However, optimal retention types for implant-supported prostheses are still being discussed [6,9,11].

There have been studies to overcome the limitations of screw- and cement-retained implant prostheses [13,14]. Shah et al. [13] reported on an abutment system that can lock and unlock prostheses by replacing cement layers with precision-machined nickel-titanium sleeves, and Lee et al. [14] reported on an implant prosthesis that could be maintained without cement by lining the surface of the crown with a composite resin. However, the research related to these prostheses is very limited, and the scientific basis is still lacking. In particular, there is no product available for partial edentulous patients among these prostheses.

On the other hand, dentists face patients with narrow ridges due to bone resorption, orthodontic therapy, missing incisors, and reduced interdental space [15]. To overcome these complicated clinical scenarios, dental implant manufacturers have developed narrow-diameter implants (NDIs) [16]. NDIs or small-diameter implants have diameters larger than mini-implants (1.8–3.0 mm) and smaller than 3.5 mm [17]. These NDIs can help avoid additional surgical procedures such as horizontal bone augmentation when bone width is insufficient, which can increase recovery time as well as medical costs and morbidity [18,19]. However, the risk for mechanical complications may increase if a single NDI is used to restore a tooth under increased occlusal force [18].

Therefore, the purpose of this study was to investigate the possibility of clinical application by evaluating the load-bearing capacity and retention characteristics of NDIs in a newly developed micro-locking prosthetic system under worst-case conditions. The first null hypothesis was that the load-bearing capacity of the micro-locking implant prosthetic system is not different from that of conventional commercialized implant systems. The second null hypothesis was that the structure modifications of the micro-locking implant prosthetic system do not affect retention, and the third null hypothesis was that retentive force remains undamaged after cyclic loading.

## 2. Materials and Methods 

### 2.1. Micro-Locking Implant Prosthetic System

**Figure 1:**
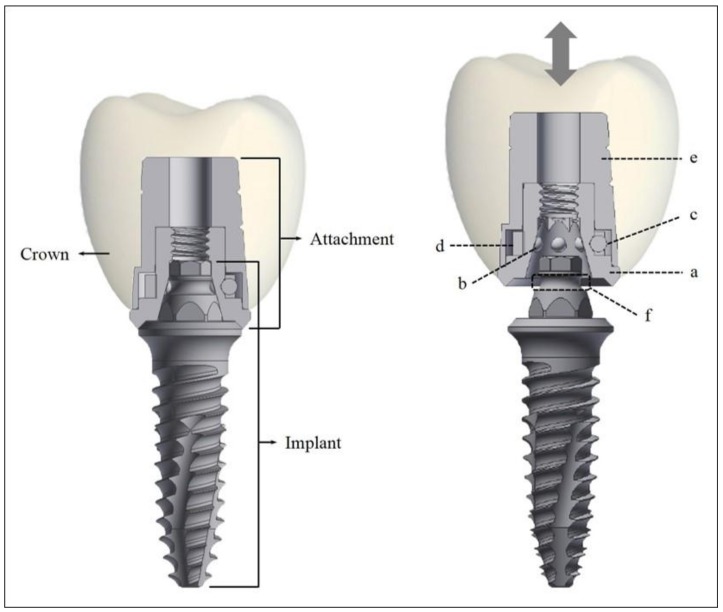
The components of the micro-locking implant prosthetic system. (a) Body; (b) ball involved in retention; (c) ball involved in preventing spring rotation; (d) spring; (e) cap; (f) retention groove.

The newly developed micro-locking implant prosthetic system consists of an assembly-type attachment and a one-piece implant (Figure 1). The attachment consists of four subcomponents: body, ball, spring, and cap. The body is as several grooved hexagonal receptacles corresponding to a hexagonal structure of the implant post, thereby preventing the rotation of the prosthesis that is attached later. The ball, the major component of zirconium oxide (ZrO_2_) and hafnium oxide (HfO_2_) (Table 1), has roughly two roles, namely (1) to participate directly in the retentive force by being seated in the retention groove and (2) to prevent the spring from rotating. The spring is located on the outside of the zirconia ball and it is mainly composed of a nickel–titanium (Ni-Ti) shape memory alloy called nitinol (Table 1). Nitinol has a shape memory effect, superelasticity, and a twinning strain [20]. Its superelasticity entails that the alloy, like a rubber object, will return to its original shape when stress is applied to the alloy and subsequently removed. When the stress is removed in this state, a reverse behavior that is opposite to the yielding phenomenon appears. In other words, its shape, despite large deformation distortion is restored while a constant stress value is maintained [20,21]. The spring used in this study, which utilizes the superelasticity of nitinol, allows the ball to be easily placed under the undercut of the retention groove in the implant post by slightly expanding when the attachment is engaged with the implant. In addition, after the attachment is engaged with the implant, the spring exerts a constant external force on the ball [21]. The cap is the part where the prosthesis is attached and has a groove and a slot to prevent the rotation of the prosthesis.

### 2.2. Load-Bearing Capacity

**Figure 2:**
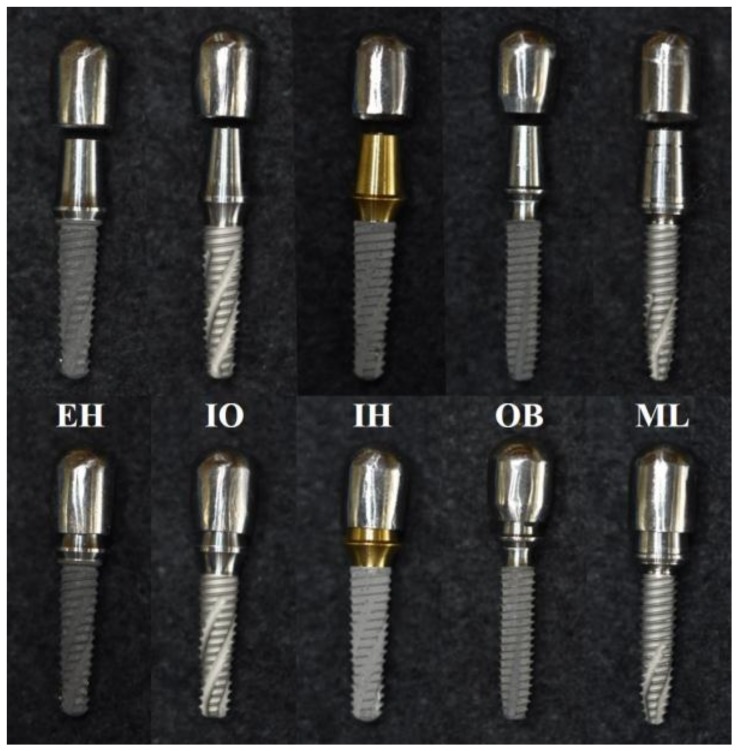
Examined implant systems (implants, abutments, and cobalt–chromium copings). EH: external-hexagon connection; IO: internal-octagon connection; IH: internal-hexagon connection; OB: one-body implant; ML: micro-locking implant system.

**Figure 3:**
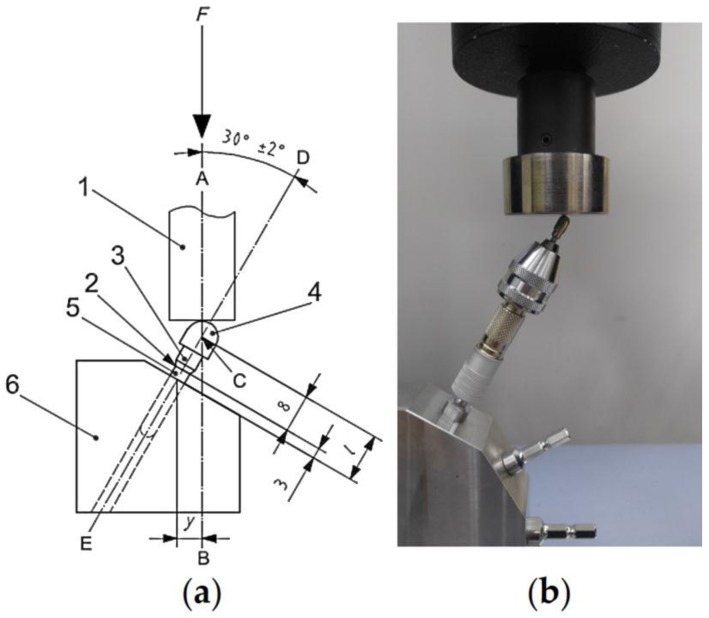
Test set-up following ISO 14801:2007. (**a**) Schematic illustration of the test design for systems with no preangled connecting part: (1) loading device; (2) nominal bone level; (3) connecting part; (4) hemispherical loading members; (5) dental implant body; (6) specimen holder. (**b**) The set-up for the mechanical testing.

Five groups were set in accordance with the implant–abutment connection designs: an external-hexagon connection (EH), an internal-octagon connection (IO), an internal-hexagon connection (IH), a one-body implant (OB), and a micro-locking implant system (ML) (Figure 2 and Table 2). Five specimens were used in each group, and the tests were conducted in accordance with the ISO 14801:2007 specifications (Figure 3) [22]. The static load test was performed at a crosshead speed of 1 mm/min using a universal testing machine (UTM) (AG-10KNX, Shimadzu, Kyoto, Japan). The load–displacement curves were recorded until either the implant assembly was visibly fractured or the displacement of the loading device reached 5 mm. After the static load test, the specimens that were not completely broken or separated were centrally sectioned along the longitudinal axis using a low speed diamond saw machine (Diamond Saw, SPG Co., Ltd., Incheon, Korea). The internal configuration was observed using an optical microscope (BX51, Olympus, Tokyo, Japan). Statistical analysis was performed using a one-way analysis of variance and a Tamhane post-hoc test (α = 0.05).

### 2.3. Effect of Structure Modifications on the Retention of the Micro-Locking Implant Prosthetic System

**Figure 4:**
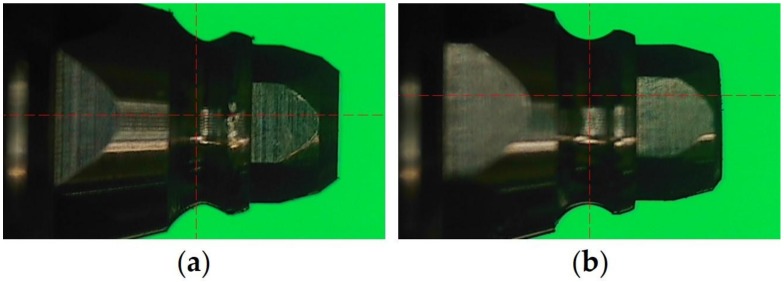
Two types of implants used in the experiment. (**a**) Implant with a non-hemispherical retention groove. (**b**) Implant with a hemispherical retention groove.

In this experiment, four groups were set depending on the structural differences of the attachment and implant as follows (*n* = 10 each group): 

(1) HB3: attachment with hexagonal receptacle and 3 balls + implant with a non-hemispherical retention groove (Figure 4a);

(2) HB6: attachment with hexagonal receptacle and 6 balls + implant with a non-hemispherical retention groove (Figure 4a);

(3) NHB6: attachment with non-hexagonal receptacle and 6 balls + implant with a non-hemispherical retention groove (Figure 4a);

(4) HB3+: attachment with hexagonal receptacle and 3 balls + implant with a hemispherical retention groove (Figure 4b).

An implant was fixed on the lower clamp of a UTM (MTS Systems Co., Eden Prairie, MN, USA). Subsequently, attachment was connected to the implant using a dedicated insert driver. A 10-cm-long metallic strap was connected to the upper clamp of the UTM, and the position of the metal chain and specimen were adjusted to generate a vertical force to dislodge the attachment from the implant. A vertical tensile force was applied to the specimen using the UTM equipped with a 1000 N load cell at a crosshead speed of 5 mm/min until the attachment was separated from the implant [8,15,23]. The peak load to dislodgment was set as the retentive force. Statistical analysis was conducted using one-way analysis of variance with Tukey’s post-hoc test (α = 0.05).

### 2.4. Effect of Compressive Cyclic Loading on the Retention of the Micro-Locking Implant Prosthetic System

To apply mechanical cyclic loading, the specimen was placed on the cyclic loading machine at 30 degrees relative to the long axis according to ISO 14801:2007 [22]. A total of 10 specimens were tested in 1,000,000, 2,000,000, 3,000,000, 4,000,000, and 5,000,000 cycles with a cyclic load of 100 N at a frequency of 2 Hz. This is equivalent to 1, 2, 3, 4, and 5 years of in vivo mastication, respectively [24]. After each cycle, unilateral removable force was applied to each specimen with the UTM (AG-10KNX, Shimadzu, Kyoto, Japan) at a crosshead speed of 0.5 mm/min at room temperature. The initial retentive force and the retentive force were measured after every 1,000,000 cycles until 5,000,000 cycles were completed, and the mean value was determined. The Wilcoxon signed rank test was used to evaluate the significance of retention loss in comparison with the retentive force in the previous cycle (α = 0.05).

## 3. Results

### 3.1. Load-Bearing Capacity

**Figure 5:**
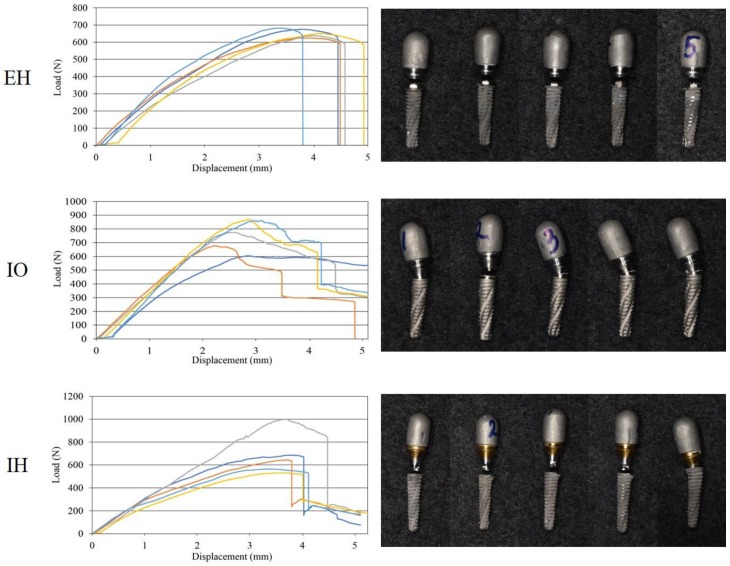

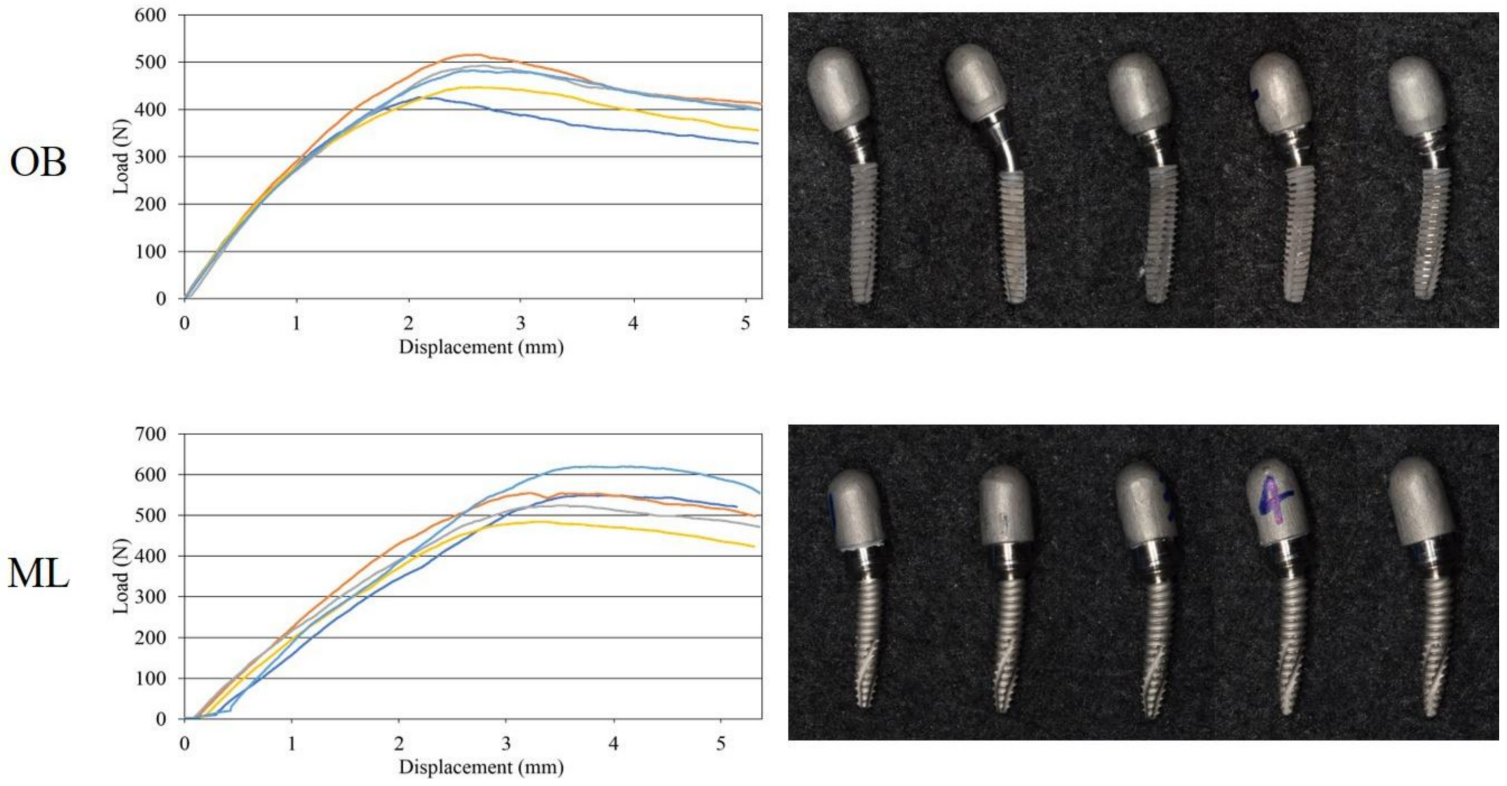
Load–displacement curves and failure mode for each experimental group. EH: external-hexagon connection; IO: internal-octagon connection; IH: internal-hexagon connection; OB: one-body implant; ML: micro-locking implant system.

**Figure 6:**
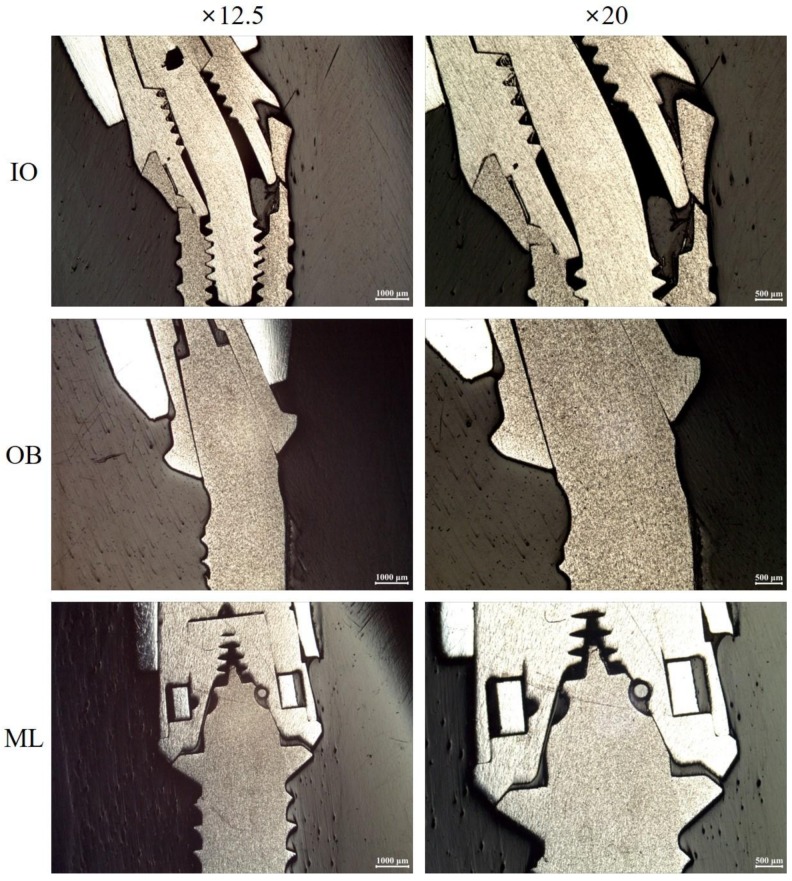
Polished cross-sections of embedded failed specimens of the tested implant systems (original magnifications ×12.5, ×20). IO: internal-octagon connection; OB: one-body implant; ML: micro-locking implant system.

The IO group showed the highest value, followed by IH, EH, ML, and OB groups (Table 3). The IO and EH groups showed significantly higher strengths than that of the OB group (*p* < 0.05), but there was no significant difference between the EH, IO, IH, and ML groups (*p* > 0.05) (Table 3). Furthermore, the IH and ML groups did not show statistically significant differences with the OB group (*p* > 0.05) (Table 3). In the EH, IO, and IH groups, most implants showed some deformation such as bending and cracks in the neck region, and abutment screws showed fracture and bending deformation (Table 4, Figure 5 and Figure 6). On the other hand, all implants in the OB and ML groups showed only bending deformations (Table 4, Figure 5 and Figure 6).

### 3.2. Effect of Structure Modifications on the Retention of the Micro-Locking Implant Prosthetic System

The raw data and the mean and standard deviation of the retentive force for each group are shown in Table 5. The HB3+ group showed the largest retentive force at 26.40 ± 2.88 N, followed by HB6, HB3, and NHB6 groups (Table 5). The HB3+ group showed significantly higher retentive forces than the HB3, HB6, and NHB6 groups (*p* < 0.05) (Table 5). There was no significant difference between the HB3, HB6, and NHB6 groups (*p* > 0.05) (Table 5).

### 3.3. Effect of Compressive Cyclic Loading on the Retention of the Micro-Locking Implant Prosthetic System

**Figure 7:**
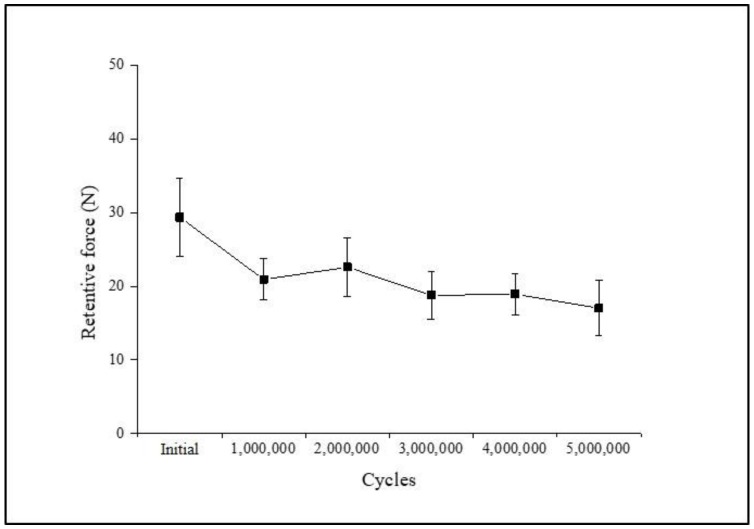
Mean retentive force as a function of the cycle number.

The mean retentive force for each cycle and the significance of the retention loss compared to the previous cycle are shown in Table 6 and Figure 7. A statistically significant retention loss was observed after 1,000,000, 3,000,000, and 5,000,000 cycles (*p* = 0.012, 0.025, and 0.025, respectively), but not after 2,000,000 and 4,000,000 cycles (*p* = 0.161 and 0.889, respectively). The retentive force decreased after 1,000,000, 3,000,000, and 5,000,000 cycles, and increased after 2,000,000 and 4,000,000 cycles. In addition, the largest change in the retentive force was observed after the initial 1,000,000 cycles among all cycles, while the smallest change was observed after 4,000,000 cycles.

## 4. Discussion

Mechanical failures of implants occur more often from the repeatable fatigue of loads less than load-bearing capacity [25]. Fatigue testing simulates in vivo conditions and thus is considered the most appropriate method of collecting data on an implant’s load-bearing capacity and lifespan [26,27]. However, static fractures also frequently develop when the load-bearing capacity of an implant is exceeded by an overload [25]. Factors that cause this type of failure include a clenching habit, ingestion of hard or coarse food, premature contact, and strong bite force [28]. Furthermore, a simple overload test can allow for conclusions about the critical regions of an implant–abutment assembly [26]. 

Numerous studies conducted under fatigue stress conditions have reported that internal connections show a load-bearing capacity and stability that are superior to those of external connections [4,29]. These results are consistent with the findings in the present study, even though different experimental methods were used. The lateral forces are effectively distributed throughout the conical interface of internal connections, but the short hexagon of the external connection is not sufficiently resistant to lateral forces [25]. This was supported by other findings showing that relatively deep and dense implant systems with force–fit connection components have a higher load-bearing capacity than implant systems with flat and joint connections [29,30]. It is also possible that the part of the abutment that is engaged in the internal connection, which is longer than that of the external connection, affect load-bearing capacity. Several studies have reported that longer joint lengths of an abutment–implant connection improve implant lifespan and load-bearing capacity [18,29,31]. Additionally, screw fracture was observed in all samples from the EH group, whereas screw fracture was observed in only one sample from the IO group. This may be due to differences in load distribution. The external force applied to the implant component of the external hexagon connection is concentrated on the abutment screw [32,33], whereas, for the internal connection, the load is distributed deeply along the inner wall of the implant and the abutment screw is protected by the long internal wall [33,34]. Although the IO and IH groups shared the same implant–abutment geometry, they showed differences in load-bearing capacity and frequency of screw fracture. The difference in wall thickness can affect the fracture resistance [23].

Compared to one-piece implants that have only shown bending deformation, two-piece implants have been shown to have various fracture patterns, including bending and cracks. Two-piece implants have greater load-bearing capacities than that of one-piece implants, which contradicts the notion that the one-body system may be stronger because it does not require a screw connection [18]. Therefore, load-bearing capacity may be significantly affected by the geometric shape of connecting parts, the length of the coupled part of the abutment, the thickness of the thinnest part of the implant collar, and the connection design [18]. Additionally, this shows that implant diameter, length, and taper may affect load-bearing capacity [35,36]. This study used two-piece and one-body implants of the same length (14 mm) with slightly different diameters (3.5 and 3.3 mm, respectively). The difference in diameter may have affected the results [29,35].

In clinical situations, a functional load of approximately 100 N is applied to the anterior teeth, and a functional load of nearly 300 N is applied to the molars, monotonically increasing the load along the dental arch [37]. However, 300 N has been suggested as an appropriate load for a single premolar implant [37], and a maximum of 700 N bite forces has been reported for the second molar region of the natural dentition [38]. Therefore, the load-bearing capacity of the micro-locking implant prosthetic system and commercialized NDIs showed sufficient strength for use in single premolar implants. 

The limitations of this study include the small number of specimens and an incomplete reproduction of actual oral conditions. Therefore, it will be necessary to imitate oral conditions realistically by reproducing complicated chewing motions in a wet environment; further research is required on a larger number of specimens and a wider range of types of implant systems.

Implant-supported prostheses may need to be retrieved when biological or technical complications occur [3,5,9]. In addition, implant-supported prostheses may sometimes have to be removed to better assess oral hygiene [39]. Accordingly, a prosthesis must have retrievability and sufficient retentive force to hold it in its place. However, due to a lack of dentistry literature, the minimum retentive force at which a prosthesis can be retrieved and kept in place is unknown [40]. The micro-locking implant prosthetic system developed in the present study used a novel approach of using a ball and spring to achieve system retention, which is completely different from any existing retention types. Therefore, there are no other studies on this topic, so the findings in the present study may provide useful information that dentists can use in actual clinical situations.

In clinical situations, the crown of a dental implant receives many different forces [23,41]. Bite force produces a combination of tensile force, compressive force, and shearing force and can induce large crown displacements, cement lute fractures, and crown breakaways [23]. However, it is almost impossible to reproduce all of these features in a laboratory [15]. The uniaxial tensile test is popular among researchers because of its reproducibility and standardization between institutions, and allows for comparisons with previously published study results [15,23]. Recently, studies have compared the retentive force of implant-supported prostheses adhered after compressive cyclic loading or mastication [40,42]. Therefore, the retentive force of the micro-locking implant prosthetic system was assessed in two experiments. The first only used the tensile test, and the second compared changes in retentive force according to cyclic loading.

In the comparison of retentive force according to structural change in the micro-locking implant prosthetic system, the HB3+ group had a higher retentive force than that of the HB3, HB6, and NHB6 groups. This demonstrates that the geometric shape of the retention groove in the implant post affects retentive force. Conversely, no significant differences in retentive force were found between the HB3, HB6, and NHB6 groups, confirming that the presence of hexagon connections or the number of balls, does not affect retentive force. The number of balls and hexagon connections may affect stability factors that provide resistance against horizontal and rotational stress, rather than the retention factors of the prosthesis.

In the comparison of retentive force of the micro-locking implant prosthetic system according to compressive cyclic loading, the maximal loss of retentive force occurred in the early loading phase (initial 1,000,000 cycles). Even in the case of cement-retained prostheses, Singer and Serfaty [43] reported that the largest loss between the adhered crown and abutment occurred during the first year. Another in vitro study found that the maximal loss of retentive force occurred in the early loading phase [24]. This is possibly due to the time required for a prosthesis to settle down and function normally in the oral cavity [44]. The retentive force of the system repeatedly decreased and increased as cyclic loading was repeated. This phenomenon may be associated with wear. Under cyclic loading, implant components may become worn out due to subtle movements, and this may lead to a loss of retentive force. However, it is also possible that changes in the implant surface due to wear increased surface roughness, and the resulting micromechanical fraction resulted in increased retentive force [45,46]. The limitation of this experiment was that there was no comparable control because there was no commercially available product similar to the micro-locking implant prosthetic system. In addition, many studies have applied an oblique dislodging load, but they were not applicable in the design of this study [47,48]. On the other hand, it is necessary to prove in further clinical studies that this retentive force is appropriate for actual clinical situations.

## 5. Conclusions

The load-bearing capacity of the NDIs of the micro-locking implant prosthetic system is similar to that of commercialized NDIs, which are mainly used in the anterior region and show sufficient strength for use in single premolar implants. On the other hand, the retention of the micro-locking implant prosthetic system was significantly affected by the shape of the retention grooves, and the retention loss rate tended to decrease after the first year and to stabilize retention over time thereafter. Studies on this novel micro-locking implant prosthetic system should be continue in various preclinical studies to verify its applicability in clinical situations.

## Figures and Tables

**Table 1:** Standard ASTM number, chemical requirements of the attachment’s components.

Components	ASTM	Chemical Composition
Body/Cap	F136 (Ti grade5)	Ti: balance
		Al: 6.07%
		V: 3.97%
		Fe: 0.15%
		O: 0.12%
		N: 0.01%
		C: 0.01%
		H: 0.0026%
Ball	F1873	ZrO_2_ + HfO_2_: 85–90%
		CeO_2_ + Fe_2_O_3_: 10–15%
Spring	F2063-03	Ti: balance
		Ni: 55.7 ± 0.3%

**Table 2:** Specifications of the tested implant systems.

Group	Implants (LOT)	Abutment (LOT)	Implant/Abutment Material	Width/Length of the Connection (mm)	Connection Type/Index	Required Torque (N/cm)	Manufacturer
EH	INNO external (16H2A) 2-piece implant, ∅3.5/14 mm	Cemented abutment (15D3A002)	Ti 4/Ti 5	4.1/0.75	External butt joint/hexagon	35	Cowellmedi Co., Ltd., Busan, Korea
IO	INNO internal (16H2A) 2-piece implant, ∅3.5/14 mm	Cemented abutment (15D4A015)	Ti 4/Ti 5	3.5/2	Internal conical interface/octagon	35	Cowellmedi Co., Ltd., Busan, Korea
IH	INNO submerged (16H2A) 2-piece implant, ∅3.5/14 mm	Cemented abutment (15D5B011)	Ti 4/Ti 5	3.35/2.9	Internal conical interface/hexagon	30	Cowellmedi Co., Ltd., Busan, Korea
OB	SlimLine (14E14-011) 1-piece implant, ∅3.3/14 mm	Cemented dual abutment (E26D04616)	Ti 4	3.5/4	Tapered external interface/cementation	-	Dentium Co., Ltd., Seoul, Korea
ML	INNO ML implant (17H1A) 1-piece implant, ∅3.3/14 mm	EZ cylinder (S17102615)	Ti 4/Ti 5	4.9/3.2	Tapered external interface with micro-locking/hexagon	-	Cowellmedi Co., Ltd., Busan, Korea Samwon DMP Co., Yangsan, Korea

EH: external-hexagon connection; IO: internal-octagon connection; IH: internal-hexagon connection; OB: one-body implant; ML: micro-locking implant system. Ti 4: commercially pure titanium grade 4; Ti 5: titanium grade 5 (Ti-6Al-4V).

**Table 3:** Mean and SD of load-bearing capacity (N) for each experimental group.

Specimens No.	Groups				
	EH	IO	IH	OB	ML
1	674.52	604.31	685.40	425.25	549.09
2	624.40	677.26	644.01	515.85	554.82
3	637.27	775.14	1000.34	492.89	524.35
4	649.97	869.01	530.65	446.63	484.25
5	681.69	861.22	564.60	482.35	619.59
Mean (SD)	653.57 (21.72) ^a^	757.39 (103.35) ^b^	685.00 (167.00) ^c^	472.60 (32.54) ^ab^	546.42 (44.24) ^d^

EH: external-hexagon connection; IO: internal-octagon connection; IH: internal-hexagon connection; OB: one-body implant; ML: micro-locking implant system. Values followed by the same letter were significantly different (*p* < 0.05, Tamhane test).

**Table 4:** Failure modes of the five experimental groups after the load-bearing capacity test (*n* = 5).

Failure Mode	Two-Piece Implant			One-Piece Implant	
	EH	IO	IH	OB	ML
**Implant**					
fracture	-	1	-	-	-
bending + crack	-	4	4	-	-
bending only	-	-	1	5	5
minor deformation	5	-	-	-	-
**Abutment/Attachment**					
dislocated	5	5	5	0	5
**Abutment screw**					
fracture	5	1	5	-	-
bending	-	4	-	-	-

EH: external-hexagon connection; IO: internal-octagon connection; IH: internal-hexagon connection; OB: one-body implant; ML: micro-locking implant system.

**Table 5:** Mean ± SD of retentive force of the four test groups.

Specimens No.	Groups			
	HB3	HB6	NHB6	HB3+
1	17.58	18.29	24.53	30.28
2	21.20	20.12	16.66	25.41
3	14.38	17.38	17.79	26.13
4	17.04	19.25	19.91	28.97
5	16.82	16.50	16.77	21.32
6	24.53	19.54	16.68	23.71
7	18.20	16.72	15.16	31.02
8	18.79	24.46	14.95	24.94
9	22.68	23.61	13.28	27.38
10	19.94	20.88	15.49	24.81
Mean ± SD (N)	19.12 ± 2.87 ^a^	19.68 ± 2.57 ^a^	17.10 ± 2.99 ^a^	26.40 ± 2.88 ^b^

HB3: attachment with hexagonal receptacle and 3 balls + implant with a non-hemispherical retention groove; HB6: attachment with hexagonal receptacle and 6 balls + implant with a non-hemispherical retention groove; NHB6: attachment with non-hexagonal receptacle and 6 balls + implant with a non-hemispherical retention groove; HB3+: attachment with non-hexagonal receptacle and 3 balls + implant with a hemispherical retention groove. Same letters indicate no significant differences between groups based on Tukey’s multiple comparison test.

**Table 6:** Mean tensile retention values at each cycle and significance of retention loss as compared with the previous measurement cycle.

Retentive Force (N)			Retention Loss (%)
Cycles	Mean ± SD	*p*	Mean ± SD
Initial	29.353 ± 5.308	-	
1,000,000	20.930 ± 2.808	0.012 *	27.705 ± 9.469
2,000,000	22.596 ± 3.982	0.161	−7.797 ± 11.057
3,000,000	18.780 ± 3.250	0.025 *	15.551 ± 13.746
4,000,000	18.924 ± 2.757	0.889	−1.535 ± 8.201
5,000,000	17.033 ± 3.720	0.025 *	10.564 ± 10.591

*p*-values are from a Wilcoxon signed-rank test with respect to the force generated for the previous measurement cycle. SD: standard deviation. * Significant difference (*p* < 0.05).
